# Ewing's sarcoma in scapula, epidemiology, clinical manifestation, diagnosis and treatment: A literature review

**DOI:** 10.1016/j.amsu.2022.103617

**Published:** 2022-04-11

**Authors:** Mohammad Nour Shashaa, Mohamad Shadi Alkarrash, Mohammad Nour Kitaz, Shahd Hawash, Mohammad Baraa Otaqy, Joudi Tarabishi, Roaa Rhayim, Hani Alloush

**Affiliations:** a Faculty of Medicine, University of Aleppo, Aleppo, Syria; b Department of Orthopedic, Aleppo University Hospital, Aleppo, Syria

**Keywords:** Ewing's sarcoma, Scapula, Epidemiology, Clinical manifestation, Diagnosis and treatment

## Abstract

**Background:**

Ewing's sarcoma (ES) can affect any bone, but its occurrence in the scapula is extremely rare. Only 15 studies investigating this condition exist in the medical literature.

**Materials and methods:**

A literature search was conducted in PubMed and Scopus, and studies on ES of scapula published in the English medical literature were retrieved. A total of 15 studies were found and were included in our study.

**Results:**

ES prevalence was highest in Asia. Moreover, ES was predominant in males (60%), with a male-to-female ratio of 3:2. ES in 53.3% and 46.6% of the cases were found in the right and left scapula, respectively. The main presentation of patients with ES of scapula was swelling, which was observed in 73.33% of the cases. Of the included studies, 46.6% used plain radiography as the primary investigation method, and 60% used computed tomography for staging and metastasis detection. For definitive diagnosis, 86.6% of the studies used immunohistochemistry markers. Adjuvant chemotherapy was considered in most studies (80%). Neoadjuvant chemotherapy was given in 6 out of 10 cases who underwent surgical treatment. Complications included malignant pleural effusion, respiratory failure, and movement restriction.

**Conclusion:**

The scapula is an extremely rare site for ES. Local invasion was found in 63.64% of the cases, whereas pre-metastases were found in 35.71% of the cases. Magnetic resonance imaging was considered to be the best radiological method used to diagnose ES of scapula. Adjuvant chemotherapy, neoadjuvant chemotherapy, and surgery were the main treatments for ES.

## Highlights

• The scapula is an extremely rare site for Ewing's sarcoma.
• local invasion was found in 63.64%, whereas pre-metastases were found in 35.71%.
• The prevalence of Ewing tumor has male predominance with male to female ratio (3/2).
• Asia has the highest Ewing's sarcoma prevalence.

## List of abbreviations

ES: Ewing's sarcoma
ESFT: Ewing's sarcoma family of tumors
PNET: peripheral primitive neuroectodermal tumor
CNS: central nervous system
ESR: erythrocyte sedimentation rate
MRI: magnetic resonance imaging
CT: computed tomography
PET/CT: positron emission tomography/computed tomography scan
FISH: fluorescence in situ hybridization
PAS: periodic acid–Schiff
BME: bone marrow examination
IEI: extra-corporal irradiation

## Sources of funding

There are no funding sources.

## Ethical approval

There is no need.

## Consent

This study does not have patients.

## Authors contribution

MNS: wrote the manuscript, data collection and conducted the literature review.

MSA: wrote the manuscript, reviewing the manuscript and corresponding author.

MNK: wrote the manuscript, data analysis and data interpretation.

SH, MBO, JT and RR: wrote the manuscript, data collection and study design.

HA: contributed to supervision, data interpretation and planning.

All authors read and approved the final manuscript.

## 1 Background

The most common primary malignant bone tumors include osteosarcoma, Ewing's sarcoma (ES), and chondrosarcoma. The scapula is an extremely rare site for primary bone tumors, with approximately only 3% of bone tumors arising from this site [1]. The ES family of tumors (ESFT) is a group of four tumors, namely extra-skeletal ES, ES of bone, Askin tumor of the thorax, and peripheral primitive neuroectodermal tumor (PNET) [2]; the use of the term “peripheral PNET” is necessary to distinguish them from nonpertinent tumors of the central nervous system (CNS) [3]. Extra-skeletal ES mainly occurs in the paravertebral area and lower extremities and rarely in upper extremities [4]. The ES of bone is a common malignant primary tumor, and it has male preponderance. ES is the second most common bone tumor in children and young adults and is the third most frequent primary sarcoma of the bone. ES accounts for 3% of all pediatric malignancies and for approximately 10% of all primary malignant bone tumors [5]. Peak incidence occurs in the second decade of life. This tumor can affect any bone, but it occurs mainly in long bones, particularly in femur, ilium, tibia, and pelvis [6]. ES rarely occurs in the skull, in the vertebra, in short tubular bones of the hands, in the feet, and in the scapula [7]. In 30% of cases, ES is multicentric in origin [8]. In 14%–50% of cases, metastases are present at the time of diagnosis [9]. In ES, CNS invasion is uncommon, and isolated CNS involvement has not been reported [10]. ES exhibits some unique features, such as proliferation of small round cells, membranous expression of CD99, and chromosomal translocations of the EWS gene on chromosome 22q12. James Ewing first described ES in 1921 as a tumor arising from undifferentiated osseous mesenchymal cells; however, it has recently been suggested that Ewing's tumor may be neuroectodermally derived from the primitive neural tissue [11]. ES is often radiosensitive, and radiotherapy can be used preoperatively or postoperatively or definitively in case surgery is not possible. The five-year survival of ES with surgery or radiotherapy alone as treatment is <10%, but survival increases to 60%–70% (in localized disease) and to 20%–40% (in metastatic disease) when surgery and multi-agent neo-adjuvant and adjuvant chemotherapy are employed in conjunction with surgery/definitive radiotherapy [12]. Compared with primary ES of the skull, skull metastases of ES are not rare, although they are uncommon based on their actual prevalence. Moreover, the location of skull metastasis of ES is unclear [13,14]. ES of scapula may present as an asymptomatic large mass with little functional deficit [1]. Localized pain and swelling are the most common symptoms of presentation [1].

## 2 Materials and methods

### 2.1 Search strategy

In July 2021, a literature search was conducted in PubMed and Scopus using the following keywords: ("Ewing's Sarcoma" OR "ewings sarcoma" OR "ewing sarcoma" OR "ewing tumor") AND "Scapula".

### 2.2 Eligibility criteria and data collection

We included the studies on ES of scapula published in the English medical literature. Those that reported other tumors of the scapula, the ES in other areas, and those involving animals were excluded. No restrictions on publication year or patient age were set. The results of all the searches were combined, and duplicated data were removed. The papers were assessed according to our inclusion and exclusion criteria, and eligibility was assessed by two authors. Fifteen cases were included in this literature review.

## 3 Results

**Fig. 1:**
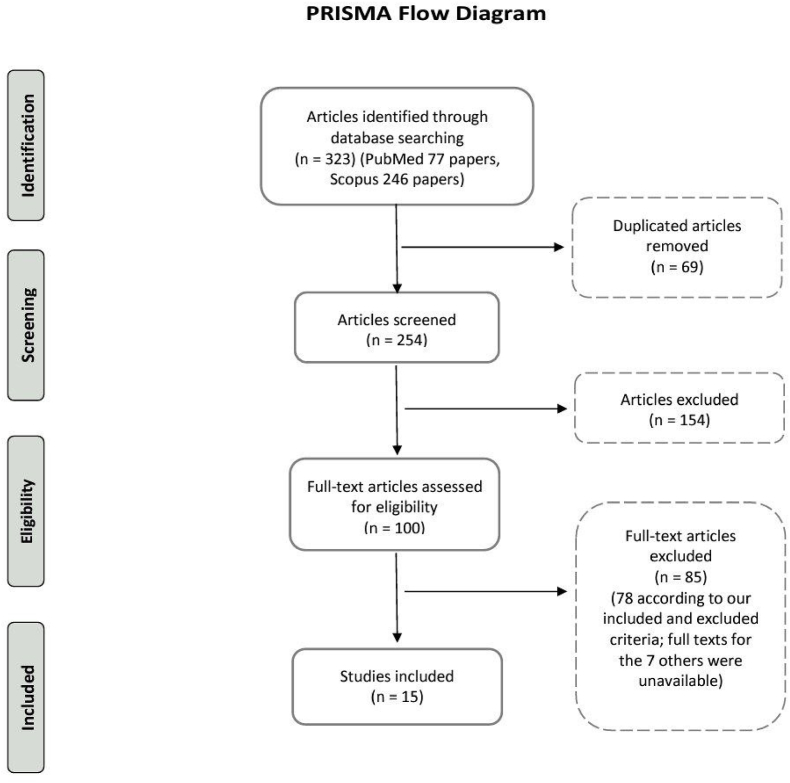
Flow chart depicting the article selection process.

In the initial search, 323 articles (PubMed: n = 77; Scopus: n = 246) were identified. After the duplicates were removed (n = 69), 254 articles were left for screening. After the titles and abstracts were screened, 100 full-text articles were retrieved, 78 of which did not meet the eligibility criteria and 7 had no available full-text. Ultimately, 15 studies were included in this study (Fig. 1).

### 3.1 Epidemiology

ES is the second most common bone tumor in children and young adults, and it mostly appears in the second decade of life [5]. In a study involving 418 patients with scapular lesions, 24 cases had ES; of these cases, 79% (19 cases) manifested the disease within the first two decades of life [15]. ES may affect any bone, especially the long bones and the pelvis, but the ES of scapula is an unusual case [16].

Of the 566 patients with bone tumor, only 9 cases (1.6%) developed a tumor in the scapula [17]. In a retrospective study on bone tumors of the scapula, out of 193 cases, only 17 cases (8.8%) had ES; the patients’ mean age was 20.5 years [18].

After searching the medical literature in PubMed and Scopus, we were able to retrieve 15 cases of ES of scapula (Table 1).

**Table 1:** Epidemiology of Ewing's sarcoma of scapula.

Number	Author	Year	Country	Age	Gender	Location	Bone or soft tissue
**1**	Hoornenborg(19)	2013	Netherlands	9 Y	M	R	B
**2**	Hosalkar(20)	2005	USA	12 Y	F	R	B
**3**	Jinkala(21)	2014	India	1 M	M	L	ST
**4**	leerunyakul(4)	2018	Thailand	18 Y	F	R	ST
**5**	Mavrogenis(22)	2009	Greece	57 Y	M	R	B
**6**	Shashaa(23)	2021	Syria	14 Y	M	R	B
**7**	Agrawal(24)	2020	India	27 Y	M	R	B
**8**	Alvegdrd(25)	1984	Sweden	15 Y	F	R	–
**9**	Asif(26)	2010	India	5 Y	M	R	B
**10**	Biswas(27)	2019	India	9 Y	M	L	ST
**11**	Chinder(28)	2019	India	24 Y	F	L	B
**12**	Hiramoto(29)	2013	Japan	65 Y	F	L	ST
**13**	Shahid(16)	2011	India	14 Y	M	L	B
**14**	Schmalzl(30)	2018	Germany	N	M	L	ST
**15**	Ralapanawa(31)	2015	Sri Lanka	25 Y	F	L	B

M: Male, F: Female, R: Right, L: left, ST: Soft tissue, B: Bone, N: neonate.

ES prevalence was highest in Asia. Specifically, ES was common in India (6/15 cases). The other Asian cases were from Syria, Thailand, Japan, and Sri Lanka. ES prevalence was second highest in Europe, which includes Sweden, Germany, Greece, and the Netherlands. Only one case was reported in the USA.

ES was predominant in males (60%), with a male-to-female ratio of 3:2. The age of diagnosis for ES ranged from neonates [19] to 65 years [20], with a median age of 14 years. Of the cases, 66.6% were aged <20 years, and 33.3% were aged >20 years; only two cases had congenital ES (Table 1).

Moreover, 53.3% and 46.6% of the cases had ES in the right and left scapula, respectively. ES originated from bone and from soft tissues in 60% and 33% of the cases, respectively. Only in one case was the origin not determined (Table 1).

### 3.2 Clinical manifestations

The main presentation of patients with ES of scapula was swelling, which was observed in 73.33% (11/15) of the cases. Swelling was associated with pain in 26.67% of the cases. In a few cases, pre-metastases [21] and lower limb weakness [22] were the main presentations. As swelling was the main and most common presentation, shoulder movement restriction was expected to be a presentation in 33.33% of the cases (Table 2).

**Table 2:** Clinical manifestations of ES.

Number	Presentation	Symptoms' history	Size	Shoulder movements restricted	Local invasion	Pre-metastatic
**1**	–	–	8*2.9*6.2 MRI	–	yes	no
**2**	Pain	–	–	–	–	no
**3**	Swelling	–	10*10*10 CT	–	yes	yes
**4**	Pain & Swelling	1 Y	26*18*13 MRI	yes	–	yes
**5**	Swelling & Pre-metastatic	–	–	–	no	yes
**6**	Swelling	2 M	9.2 × 7.7 × 7.6 MRI	yes	yes	no
**7**	Pain & Swelling	1 Y	–	no	no	no
**8**	–	–	–	–	–	–
**9**	Pain & Swelling	5 M	–	no	yes	yes
**10**	Swelling	2 M	8 × 9 CE	yes	yes	no
**11**	Swelling	2 M	18 × 27 × 26 MRI	yes	yes	no
**12**	Swelling	–	–	–	no	no
**13**	Pain & Swelling	15 D	1 × 0.7 × 0.7 CE	yes	yes	no
**14**	Swelling	–	4 × 3 CE	no	–	no
**15**	lower limb weakness	1 D	–	–	no	yes

MRI: Sizes were obtained through magnetic resonance imaging, CT: Sizes were obtained through computed tomography scanning, CE: Sizes are taken during clinical examination.

Various pain characteristics were reported, such as pain that worsens when one moves or that intensifies at night [4,23], and persistent pain unrelated to posture [16] with varied responses to antipyretics and analgesics. Systemic symptoms, such as weight loss, fatigue in 6.67% of cases [4], and fever in 13.33% of cases [4,23] were uncommon complaints. Such laboratory findings as anemia, leukocytosis, and elevated erythrocyte sedimentation rate (ESR) were also noted [24]. The time period between the onset of the initial symptoms and the diagnosis varied among the patients, ranging from 1 day to 1 year, with a frequency of 2 months (Table 2).

As regards physical examination results, 53.33% of the cases showed swelling with a hard to firm consistency in the scapular region. In general, no neurovascular deficits were reported. One case unexpectedly developed neurological retrogradation that involved the upper limb 3 days after the onset of symptoms due to severe cord compression caused by intradural extramedullary mass with an extra-spinal component [22]. Tumor size was determined through radiographic investigations, such as magnetic resonance imaging (MRI) and computed tomography (CT). Also, tumor size was determined during clinical examination in 20% of the cases [16,19,25]. The largest reported tumor size was (26*18*13 as determined using.

MRI) [4]. Tumor size was not reported in 6 out of the 15 studies (Table 2).

As ES develops rapidly, local invasion is predictable. Local invasion was found in 63.64% of the cases, and premetastases were observed in 35.71% of the cases. Metastases were observed in the lungs (4/15) [4,20,21,26], lymph nodes (1/15) [4], bone marrow (1/15) [27], skeleton (1/15) [28], and spinal intradural (1/15) [22].

### 3.3 Diagnosis

ES diagnosis involves imaging, histopathological examination, and immunohistochemistry markers, which provide a definitive diagnosis. The main imaging investigations performed were as follows: Plain radiography was used in 46.6% of the studies as the primary investigation method, which revealed ES as a lytic or sclerotic lesion in addition to being a soft tissue mass. CT scanning was used in 60% of the studies either for staging and metastases detection or for the initial investigation of the tumor, which may appear as heterogenously enhancing soft-tissue mass, osteolysis of bones, or cystic changes. MRI was used in 73.3% of the studies for ES evaluation, which revealed the tumor as lobulated heterogeneously expansile lytic lesion with T1 isointensity and T2 heterogeneous high-signal intensity; in one study, MRI showed multiple fluid levels, degenerative cystic changes, hemorrhage, and necrotic areas [29]. MRI is considered to be the best radiological method used to diagnose ES of scapula. As for metastasis detection, positron emission tomography (PET)/CT scan (26.6%) and bone scintigraphy (26.6%) were the tools that were primarily used. In the PET/CT scan, ES appeared as a lytic expansile lesion, and the metastases were seen as abnormal hypermetabolic enhancing masses. Bone scintigraphy revealed an increased uptake in the ES sites (Table 3).

**Table 3:** Diagnosis of Ewing's sarcoma.

Number	Radiograph	MRI	CT scan	immunohistochemistry	PET Scanning	Bone scintigraphy	Other Tests
**1**	No	yes	yes	no	no	yes	no
**2**	yes	yes	yes	yes	no	yes	no
**3**	no	no	yes	yes	no	yes	US + BME
**4**	yes	yes	no	yes	yes	no	FISH
**5**	yes	yes	yes	no	no	no	no
**6**	no	yes	yes	yes	no	yes	no
**7**	yes	yes	yes	yes	yes	no	no
**8**	–	–	–	–	–	–	–
**9**	yes	no	no	yes	no	no	no
**10**	no	no	yes	yes	yes	no	no
**11**	no	yes	yes	yes	yes	no	no
**12**	no	yes	no	yes	no	no	FISH
**13**	yes	yes	no	yes	no	no	no
**14**	no	yes	no	yes	no	no	no
**15**	yes	yes	yes	no	no	no	no

US: ultrasound scan, FISH: fluorescence in situ hybridization, BME: bone marrow examination.

One study (6.6%) used ultrasound imaging to evaluate ES, which appeared as a heterogenous solid lesion with multiple echogenic foci [27], indicating that ultrasound imaging is less suitable for ES diagnosis. Fluorescence in situ hybridization (FISH) was used in 13.3% of the studies to confirm EWS gene translocation [4,20]. Histological examination showed clusters of small blue round cells with round or ovoid nuclei, distinct nuclear membrane, powdery chromatin, and occurrence of mitosis. Rosette and pseudo-rosette were found in one case [30]. Another study performed periodic acid–Schiff (PAS) staining, which revealed the presence of abundant cytoplasmic glycogen [16]. Histologically, ES may be misdiagnosed with small cell osteosarcoma, mesenchymal chondrosarcoma, lymphoma, metastatic and primitive neuroectodermal tumor (PNET) [16].

For definitive diagnosis, 86.6% of the studies used immunohistochemistry markers, including CD-99, vimentin, and FLI-1 primarily. In addition, one study (6.6%) performed bone marrow examination (BME), which revealed the infiltration of atypical cells [27].

### 3.4 Treatment

Generally, chemotherapy, surgery, and radiotherapy are the treatment approaches for ES [31]. Specifically, adjuvant chemotherapy and neoadjuvant chemotherapy play an important role in ES treatment. Adjuvant chemotherapy was considered in most of the studies (12/15).

In one of the three remaining studies, no chemotherapy was given because of the rapid metastasis of the disease that led to death prior to treatment initiation [22]. In the two other studies, neoadjuvant chemotherapy was considered [21,30]. Neoadjuvant chemotherapy was given in 6 of the 10 cases who underwent surgical treatment (Table 4).

**Table 4:** Treatment of Ewing's sarcoma.

Number	Surgery	Adjuvant chemotherapy	Neoadjuvant chemotherapy	Radiotherapy	Complications	Prognosis
**1**	yes	yes	no	Yes	restriction	alive
**2**	yes	yes	yes	No	no	alive
**3**	no	yes	–	–	no	–
**4**	yes	yes	yes	Yes	metastasis and local recurrence	dead
**5**	yes	no	yes	No	no	alive
**6**	no	yes	no	No	–	–
**7**	yes	no	yes	No	no	alive
**8**	no	yes	no	Yes	metastasis	–
**9**	no	yes	no	Yes	–	alive
**10**	yes	yes	yes	Yes	local recurrence	–
**11**	yes	yes	no	Yes	no	alive
**12**	yes	yes	no	no	malignant pleural effusion	dead
**13**	yes	yes	no	no	no	alive
**14**	yes	yes	yes	no	metastasis	alive
**15**	no	–	–	–	respiratory failure	dead

The most common chemotherapy medications were vincristine, ifosfamide, cyclophosphamide, doxorubicin, etoposide, actinomycin D, adriamycin, and melphalan. Several protocols were applied, such as the Ewing 99 protocol [32], the Rosen's T-2 protocol [26], the modified St. Jude's protocol [25], and the Cooperative Waeichteilsarkom Studiengrappe protocol [19]. Some studies did not provide details of the chemotherapy treatment applied. The addition of ifosfamide and etoposide to the treatment regime improved the outcome in nonmetastatic ES patients [33].

Only 6 out of the 15 studies used radiotherapy. Intraoperative extra-corporal irradiation (IEI) was used by Hoornenborg et al. due to poor functional results obtained with scapular prosthesis surgery [32]. Postoperative radiotherapy improved local outcomes, with marginal resections, close surgical margins, poor response to chemotherapy, or large tumor sizes [1,32,34].

Surgery accompanied by neoadjuvant chemotherapy has higher survival outcomes compared with chemotherapy and/or radiotherapy alone [1]. All patients without metastases were treated surgically, except in Alvegdrd et al.’s study, wherein the patient had developed single pulmonary metastasis [26]. Three cases [22,27,28] were not treated surgically due to metastases, but Mavrogenis et al. performed surgery despite the occurrence of metastasis, and the one-year follow-up outcomes were normal [21]. The main surgical procedure was scapulectomy. Mavrogenis et al. considered constrained reverse total shoulder reconstruction following scapulectomy [21]. Leerunyakul et al. excised the tumor while preserving the glenohumeral joint [4]. Hoornenborg et al. performed intraoperative extra-corporal irradiation [32]. The other studies did not report the details of the tumor excision performed. For patients who underwent surgery, the survival rate was 86.5% at 5 years and 81% at 10 years. The total survival rate was 71.4% at 5 years and 63% at 10 years for all patients [1].

Alvegdrd et al. treated the metastatic disease using chemotherapy and segmental underlobectomy, but they did not provide follow-up data [26]. Jinkala et al. also gave conservative treatment for the metastatic disease, but no follow-up data were provided [27]. Meanwhile, intensive chemotherapy could control single pulmonary metastasis [31].

Complications included metastases, local recurrence, malignant pleural effusion, respiratory failure, and movement restriction. (As indicated in Table 4, three patients died due to ES, but most studies mentioned only a few details about the follow-up, so we cannot report the actual mortality rate). Nevertheless, the survival rate was not affected by local recurrence [1].

## 4 Conclusion

The scapula is an extremely rare site for ES. ES prevalence was highest in Asia, and it had male predominance (60%), with a male-to-female ratio of 3:2. The main presentation of patients with ES of scapula was swelling, and systemic symptoms were uncommon. As ES develops rapidly, local invasion was found in 63.64% of the cases, whereas premetastases were found in 35.71% of the cases. Plain radiography was used in 46.6% of the studies as the primary investigation tool, and MRI was considered the best radiological method used to diagnose ES of scapula. Adjuvant chemotherapy, neoadjuvant chemotherapy, and surgery were the main treatments for ES.

## Provenance and peer review

This study is not commissioned, and it has been externally peer reviewed.

## Declaration of competing interest

The authors declare that they have conflicts of interest.
